# The impact of self-harm by young people on parents and families: a qualitative study

**DOI:** 10.1136/bmjopen-2015-009631

**Published:** 2016-01-06

**Authors:** Anne E Ferrey, Nicholas D Hughes, Sue Simkin, Louise Locock, Anne Stewart, Navneet Kapur, David Gunnell, Keith Hawton

**Affiliations:** 1 University Department of Psychiatry, Centre for Suicide Research, University of Oxford, Oxford, UK; 2 School of Healthcare, University of Leeds, Leeds, UK; 3 Health Experiences Research Group, Nuffield Department of Primary Care Health Sciences, University of Oxford and NIHR Oxford Biomedical Research Centre, Oxford, UK; 4 Central Oxon CAMHS, Oxford Health NHS Foundation Trust, Oxford, UK; 5 Centre for Suicide Prevention, University of Manchester and Manchester Mental Health and Social Care Trust, Manchester, UK; 6 School of Social and Community Medicine, University of Bristol, Bristol, UK

**Keywords:** MENTAL HEALTH, QUALITATIVE RESEARCH

## Abstract

**Objectives:**

Little research has explored the full extent of the impact of self-harm on the family. This study aimed to explore the emotional, physical and practical effects of a young person's self-harm on parents and family.

**Design and participants:**

We used qualitative methods to explore the emotional, physical and practical effects of a young person's self-harm on their parents and family. We conducted a thematic analysis of thirty-seven semistructured narrative interviews with parents of young people who had self-harmed.

**Results:**

After the discovery of self-harm, parents described initial feelings of shock, anger and disbelief. Later reactions included stress, anxiety, feelings of guilt and in some cases the onset or worsening of clinical depression. Social isolation was reported, as parents withdrew from social contact due to the perceived stigma associated with self-harm. Parents also described significant impacts on siblings, ranging from upset and stress to feelings of responsibility and worries about stigma at school. Siblings had mixed responses, but were often supportive. Practically speaking, parents found the necessity of being available to their child often conflicted with the demands of full-time work. This, along with costs of, for example, travel and private care, affected family finances. However, parents generally viewed the future as positive and hoped that with help, their child would develop better coping mechanisms.

**Conclusions:**

Self-harm by young people has major impacts on parents and other family members. Clinicians and staff who work with young people who self-harm should be sensitive to these issues and offer appropriate support and guidance for families.

**Strengths and limitations of this study**

- This study included interviews with a large sample of 37 parents who explained their experiences with their son or daughter's self-harm.
- Parents from a range of backgrounds and geographical areas in the UK were included.
- A thematic analysis was conducted independently by three researchers and any differences of interpretation were resolved by discussion.
- Most participants were mothers and diversity was limited, with one participant from a non-Caucasian ethnic background.

## Introduction

Self-harm (ie, intentional self-poisoning or self-injury) is reported by at least 10–15% of adolescents in the UK^1^ and by substantial proportions of adolescents in other countries.^2^ ^3^ Considerable research has examined self-harm in terms of the characteristics, intentions and outcomes of the individuals involved.^4^ Initial research on self-harm and parenting focused on family dynamics that might contribute to a young person's self-harming behaviour. Family/environmental factors such as childhood abuse, pathological family relationships and parent–child discord have been found to be risk factors for self-harm,^5–7^ as has a parent's previous self-harm,^8^ but most young people self-harm as the result of a complex combination of experiences.^9^ However, a young person's self-harm can have significant impacts on the parents and family.^10^ Parents describe feelings of shock, disappointment, guilt, fear, frustration and anger,^11^ ^12^ but there has been limited investigation of the practical impact of self-harm on the lives of families^13^ and how this affects parenting strategies.^10^ Siblings’ experiences have received very little research attention, focusing mostly on parents’ worries about sibling neglect.^13^ ^14^

We conducted an in-depth qualitative study with parents of young people who self-harmed to determine the emotional and practical impacts of their child's self-harm. We explored the impact of the self-harm on the wider family, including siblings, and examined parents’ views of their child's future. The overall aim was to generate information that could be helpful for parents and families and to highlight their needs for those providing care.

## Methods

### Sample and recruitment

We conducted semistructured narrative interviews with 37 parents of 35 young people who had self-harmed (including two parent pairs). A further three people were interviewed but later withdrew from the study, and one person whose husband self-harmed was not included in this analysis. Participants were recruited through mental health charities, support groups, clinicians, advertisements, social media, personal contacts and snowballing through existing contacts. Clinical teams and organisations with members who might have been interested in participating were given information about the study to pass on to them, in person or via social media. Individuals who expressed an interest in participating received an introductory letter, a detailed information sheet and a form to return in a prepaid envelope if they wished to participate. Participants were contacted by telephone or email and encouraged to ask questions about the study, and an interview was arranged in a location of their choosing.

We sought a maximum variation purposive sample^15^ ^16^ in order to capture a wide range of experiences. We aimed for variation across demographic characteristics including gender, ethnicity (while recognising the difficulties this can present^17^), and geographical location (with a planned focus on Oxfordshire and Buckinghamshire for recruitment via clinicians).

### Data generation and analysis

Participants were interviewed between August 2012 and October 2013 for an average of 84 min. Interviews were video or audio recorded and began with an open-ended section in which the participant explained their experiences of caring for a young person who self-harmed without interruption. The interviewers then asked follow-up questions to get more information about the participant's story. Finally, we used semistructured prompts based on topic areas identified through a literature search and suggestions from the project's Advisory Panel (which included parents, researchers and clinicians). Sixteen people were interviewed by NDH and 21 by SS, both experienced interviewers.

The interviews were transcribed verbatim from audiotapes by professional transcribers and carefully checked by the researchers. Participants could remove any part of the interview before giving their written consent for the content to be used in research and for publication on http://www.healthtalk.org, where a summary of findings are available. Final transcripts were uploaded to NVivo V.9 for initial coding by NDH and SS. A coding framework of anticipated and emergent themes was developed using constant comparison techniques. Data were assigned to categories using the NVivo ‘node’ function, based on close reading and interpretation of the interview transcripts. Coding reports were generated and used for an initial overarching thematic analysis.^18^ Broad themes were then identified based on the summary of all the issues raised by participants on particular topics. Two researchers (NDH and SS) conducted this analysis independently and resolved any discrepancies or differences of interpretation through discussion, using regular peer debriefing and reflexive conversations between the researchers.^19^ A more focused analysis on the themes relating to the impact of self-harm on families was then conducted by AEF using QDA Miner Lite 4 software.

Participants gave informed written consent before their interview. Pseudonyms were assigned to all participants to ensure confidentiality and anonymity. The study was approved for national recruitment by the Berkshire Research Ethics Committee (09/H0505/66).

## Results

Interviewees lived in England, Scotland and Wales (table 1). Twenty-nine of the young people who self-harmed were daughters and six were sons (table 2). Over two-thirds were aged under 16 years at the time of their first episode of self-harm (average age 15.1 years). For nearly all the young people, self-harm included self-cutting, but over half had taken overdoses; other methods included, for example, burning and strangulation. All had engaged in multiple acts of self-harm, although there was a range of severity in terms of physical danger and some were no longer self-harming at the time of the interview. Some of the young people had mental health problems, including eating disorders (which parents often saw as a form of self-harm).

**Table 1:** Demographic characteristics of the parents and carers

		N	
Relationship to young person			
Mother		32 (1 adoptive)	
Father		5	
Ethnicity			
White		36	
Black British		1	
Geographical location			
Southeast	5	Southwest	3
Northern England	2	East	1
Oxon and Bucks	14	Wales	2
Midlands	6	London	2
Scotland	2		

**Table 2:** Demographic characteristics of young people who self-harmed

	Females (years)	Males (years)
	N=29	N=6
Average age started self-harm	13.8	16.3
Range	9–20	9–21
Average age at time of interview	18.7	22.8
Range	14–24	17–28

### Immediate impact

Parents discovered their child's self-harm in different ways. Some found out from teachers, their children or friends of their children. Others had suspicions and searched for information (eg, reading the child's diary). Nigel and his wife discovered their daughter's self-harm when the police arrived one night after an alert from a child helpline. “To the best of my knowledge [she was] asleep in bed, [but she] had been in touch with one of the agencies… and … was talking about self-harming.” Discovery of self-harm can be very distressing for parents, particularly when an injury or overdose necessitates hospital treatment.

Many parents characterised their initial reaction as ‘shock and horror’. Shannon said, “First feeling was my stomach dropped and I felt sick and I thought, oh no, it’s really that bad.” Isla also felt ‘frustrated… annoyed… and angry’ that her daughter had hurt herself, and Amber said, “at first, when you see these marks on your child’s beautiful skin, you're just filled with every emotion that you can possibly think of—fear, anxiety, disbelief, anger and just not knowing what to do.”

### Ongoing impact on parents’ emotional state and mental health

Parents described considerable stress and anxiety caused by their child's behaviour. Jocelyn said, “I couldn't stop crying… I was really upset, couldn’t sleep.” Rebecca described the experience as ‘chaotic’ for everyone in the house, and Judith was “surprised [she] never got carted off in a white jacket.”

Several parents reported feelings of guilt, shame or embarrassment associated with their child's behaviour. Louise said, “If I'm honest with myself, there is an element of shame because it's almost like a weakness. Why could I not help her more?” Similarly, Nancy said, “I don’t want [other mums] to know because I feel ashamed of what she's done and I feel responsible for it.” Susanne said, “Everyone is really embarrassed. I’m embarrassed by it, you know, because you think you've failed because if they were normal, well-balanced children they wouldn't be doing these things.” Some parents, however, refused to feel ashamed about their child's self-harm.

Depression was common among the parents, which some related directly to their child's self-harm. As the self-harming behaviour continued, parents reported becoming worn down.

I'm tired. Emotionally, I'm so tired and I want it to stop and, whilst I would never commit suicide, the thoughts are there at times, you know. I have actually pre-planned what I would do and how I'd do it. So it does have a knock-on effect… And the depression it leaves with you is very hard because you're almost constantly living a lie. (Flora)

Two parents reported having themselves self-harmed in the past. Their daughters’ self-harm precipitated complicated feelings of guilt and responsibility for their daughters’ behaviour. In Joy's case this resulted in a relapse of her own self-harm. She had worked hard to stop self-harming, but returned to this method of regaining a sense of control in her life.

The stress of coping with their child's problems caused physical symptoms in some parents. When Georgia discovered her daughter's self-harm, she felt ‘sick, really, physically sick’. Alana felt ‘jumpy and fearful’ and lost a lot of weight. Janet suffered from panic attacks, chest pains and physical exhaustion. Often sleep was disturbed, due to anxiety or the need to be vigilant about the child's movements at night. Some parents reported sleeping near their children to ensure they were safe—for example, on the floor beside the bed or outside their door.

Participants varied in their method of coping with their stress, anxiety and mental health problems. Some were prescribed antidepressants, while others used counselling, cognitive behavioural therapy or mindfulness training. Most parents felt that only by taking care of themselves could they help their children, although several did not seek help.

### Impact on partners

The stresses associated with a young person's self-harm can affect relationships between family members, sometimes leading to marriage difficulties. Nadine's daughter's mental health issues and self-harming behaviour ‘put my marriage under a colossal amount of strain’, while Sally and her husband had to have separate holidays to cope with the stress. Jacqueline thought that parents’ relationships had to be put on the back burner while coping with a child's self-harm. “You are just putting life on hold until this is sorted out. Because if you try to make demands on each other during the middle of this, you're not going to survive it.” In a similar vein, Jocelyn didn't tell her partner ‘half the things’ her daughter did, because she felt it would be too much for him and perhaps for the relationship.

### Impact on siblings

Siblings’ reactions to the discovery of self-harm varied. The disruption in the family could be very difficult, especially if it happened around an important time like school examinations, and parents worried about balancing the needs of all their children. Some siblings became extremely upset or angry: Sally's younger daughter had fits of rage, and Ellen's son was angry and verbally abusive towards his sister. Theresa's daughter reacted to her brother's behaviour with resentment, anger and frustration. “She resents the amount of attention that it's warranting and the focus and the anger. I mean she’s seen a lot of upset and anger… that she feels are all caused by her brother.”

Some siblings were extremely supportive. Evelyn's step-daughter made a long drive every week to attend family therapy and Louise's daughters became particularly close because of their shared mental health problems. Some siblings had conflicting responses: Isla's older daughter “could see that that her sister was suffering and so on one hand she was incredibly sympathetic and wanted to be supportive and on the other, quite irritated by what she'd done.” Usual sibling behaviour (eg, teasing and squabbling) could become fraught, but in other cases it was taken as a sign that everything was ‘normal’. Some siblings became more overtly supportive or protective of their brother or sister. Vanessa's son was very concerned about his sister after her self-harm, but felt awkward moving too far away from their sibling roles.

He went to have a little chat with her and, normally he just takes the mick out of her, [a] normal annoying older brother… And he just went up to say, “I’m here for you if you need me. I love you very much and no matter how much of this banter we have between us, I’m here for you.” And he came down, after talking to her…'I don't know how long I can keep this sort of thing up. I need to go back to being the annoying brother.’

Siblings may feel responsible—trying to avoid irritating their brother or sister lest they self-harm. Charles’ daughter had to “bite her tongue… and feels very constrained about what she can say or do with her brother [lest he self-harm]… it's a great worry for her.” Shannon's son had to come to terms with his sister's self-harm:

He felt quite upset that he hadn't been there for her. Being the older brother, he should have been there to look out for her… I explained that… he can he can be there for her now and he is. He’s very supportive and goes to see her a lot now. Still carries on with the usual jokes and winding each other up but that’s normality for her so it's good.

School-age siblings, especially when close in age, may feel the stigma of a sibling's self-harm. For example, Denise's son did not want his friends to know about his sister's self-harm. “There’s been a big issue because he’s just kept the whole thing secret so none of his friends know. He doesn’t talk about it because he thinks that people will bully him [by saying], ‘Oh your sister self-harms’.”

In some cases, family relations become so strained that some siblings removed themselves from the family altogether.

### Impact on wider family

Relationships with parents (ie, the young person's grandparents) could be affected. The crisis in the family often had the effect of bringing pre-existing problems in relationships to the fore. However, some grandparents were determined to help—for instance, Amy's daughter's grandparents got books out of the library to help understand the problem.

Parents worried about family members overreacting or getting upset, as well as about judgment and blame. Joy and Nadine were both told by family members that they should ‘sort [your] daughter out’. Flora's family said, ‘You made your bed—you lie in it’, which Flora thought was not supportive given the magnitude of her son's problems.

Some parents felt trapped in the middle—coping with their child's problems and their parents’ problems (including ill-health) at the same time. Ill-health could be made worse by emotional reactions to finding out about a grandchild's self-harm. Paul's mum became very ill on hearing about her granddaughter's self-harm, and Janet's elderly mother was ‘heartbroken’ when she found out, and the stress and worry exacerbated her medical conditions.

Some extended families were supportive. Jocelyn said her parents had been ‘brilliant’. Evelyn's family were so supportive when her daughter went into hospital, despite their shock, that she felt that the overall effect was to bring the family together.

### Social isolation and social support

An overall theme was a profound sense of isolation and a desire to keep a child's problems private. This was often linked to parents’ feelings of guilt and their worries about what others might think. According to Joan, “It can be very lonely… you can tell everybody but people will then cross the road to avoid talking to you.” Georgia withdrew from clubs and groups rather than pretending everything was fine. Similarly, Julian and his wife stopped socialising to avoid answering questions about their daughter. Over time, this type of social avoidance could lead to the temporary or permanent loss of friends.

Secrecy surrounding self-harm can add to the strain on the child and the family. Jacqueline felt that secrecy increased the pressure on her daughter and became a problem in itself. “The trouble is, the longer you go on not being open, the harder it is to deal with it, and the more confusing it is for the people around you.”

Conversely, friendships could function as important sources of support, especially friends who also had experience of a relative's self-harm or psychiatric problems. For example, Julian kept in touch with another father whose daughter had similar problems for mutual support. Several parents indicated that they might prefer a support-group setting, where the other participants were both strangers (no risk of sensitive information being passed on to family or friends) and people who had similar experiences. Hearing stories about self-harm from others was helpful for several parents. Vanessa said, “Just hearing other people’s stories makes you feel like you're less alone…you can gain a lot of strength from that.”

### Impact on work and finances

Many parents reported that their child's self-harm had a detrimental effect on the family's financial situation, often by making it difficult for parents to maintain a full-time job. Parents’ desire to be available when their child needed them—including being able to leave work at short notice—often conflicted with the requirements of employment. Flora said, “My boss is very good but there comes a limit. They’ve got criteria to fulfil as well and you can’t just keep spinning off… my mind's whirling, you know, am I going to get a call?”

Other parents took leave, paid or unpaid, to care for their children, sometimes at short notice. Some parents decided to give up work entirely to focus on their children, including Denise. “I’ve took a career break for a year so I can be at home… we needed someone about.” Georgia “chose not to work because I didn't want to go out… If I did have to go out, I was fearful [about] what I would find when I came home.”

Self-employed parents particularly struggled, as the household income depended entirely on their ability to work. Paul was self-employed but wanted to be available when his children returned from school, but worried that this would cost him future business because he had to postpone jobs.

Parents often spent considerable amounts of money on private psychiatric care or counselling for their child. Some also paid for private tutoring when their children were unable to attend school due to hospitalisations or crises. Travel costs could be extensive when children were placed in far-away inpatient care or had self-harmed in another country. Some parents bought treats for their children to ‘compensate’ for the impact on their lives. The inability of older children to move out of the family home and support themselves caused additional financial strain in some cases.

### Parents’ conception of the future

Parents’ thoughts about the future were mostly guardedly positive. Some were very optimistic, but others were afraid to express optimism in the face of an uncertain future for their child. For example, Julian “[couldn't] even consider life beyond this because I just don’t know what the reality is going to be.” Theresa felt she ‘almost daren't hope’ that her child could recover and Flora said that she would ‘always be wary’ because of her son's past behaviour. A common theme was ‘taking life one day at a time’.

Parents were aware of their child's vulnerabilities and concerned about their ability to cope as an adult. They worried about the effect the stigma of self-harm might have on others’ opinions of their child. However, parents expressed hope that as the child matured, they would develop more appropriate coping mechanisms, perhaps with the help of professionals, and would move on to a happy adulthood free of self-harm.

Many parents saw their future role as supporting a more independent child. Denise said that “seeing people that have been through it and come out the other side … gives you… hope.” Indeed, some parents whose children had self-harmed in the past had become less likely to harm themselves with age. However, some parents felt they would struggle to move on—Evelyn said, ‘I'm stuck in the trauma’.

Overall, parents described a sense of hope for the future, combined with an understanding that their child might be vulnerable to life's difficulties. According to Nadine,

I see the future as being a little bit like a contour map in that she will continue to get better and she will have long periods where life is good… and where her coping strategies are healthy and productive and meaningful. And then I see that there will be times… where she will cope less well and I see troughs at the bottom, where she may well resort to hurting herself… The older she gets, the more experienced she gets at dealing with stuff and coping with stuff and learning how to deal with her emotions better.

## Discussion

This study shows that self-harm can have extensive impacts on families. Previous research has largely focused on parental emotional responses to the discovery of a child's self-harm.^20^ We have shown that self-harm by young people can not only have extensive effects on parents’ emotional states, but also on their mental health, relationships with partners, children, their parents and friends, and on work and finances.

Parents’ emotional states and mental health were often negatively affected by the difficulty of making sense of self-harm.^21^ Stress and anxiety related to the self-harm lead to negative emotions and in some cases to depression and physical symptoms, including sleeplessness and weight loss. Relationships with partners could also be affected: marriages became strained and sometimes communication was disrupted. A child's self-harm may particularly affect siblings, who are often similar in age and may be struggling with their own journey through adolescence or young adulthood. They are likely to be affected by family upset associated with a young person's self-harm. Sibling reactions included anger, resentment and frustration, as well as the desire to help and support their brother or sister. As in previous studies of mothers, parents reported that time, energy and attention spent on the child who had self-harmed led to neglect of the other children,^13^ ^14^ due to lack of time and resources. Similarly to previous studies,^11^ ^20^ parents worried about doing or saying something that might precipitate an episode of self-harm; siblings had similar worries that their ‘irritating’ behaviour might lead to further self-harm.

Parents’ relationships with their own parents could be negatively affected. Some older family members did not understand self-harm and refused to discuss it. In extreme cases, the shock exacerbated illness. However, self-harm in the family could also strengthen relationships. Grandparents who were able to engage instead of judging sometimes developed stronger relationships with their children and grandchildren. Some parents became socially isolated due to their perceived stigma of self-harm. When parents were able to discuss the self-harm with friends, however, this could lead to a stronger support network. Some parents felt that it might be easier to talk to strangers, especially in the context of a support group. Parents felt that hearing about the experiences of others might help them cope.

Many parents described feelings of shame and guilt. These reactions were often related to the stigma they felt was attached to self-harm and mental illness. A common worry was that the self-harm was a result of something they did or did not do as a parent. Parents were also afraid that their family and their parenting would be judged by others, and that the stigma of self-harm might have a negative effect on the child's future.

### Strengths and weaknesses

The study benefited from inclusion of a larger sample of parents than other studies. While it included both mothers and fathers, most participants were mothers, reflecting the difficulty of recruiting fathers for such research.^22^ However, mothers frequently talked about their partners’ reactions to the self-harm. Because we only spoke to parents, we can only report their interpretation of the impact on their children and family members. While the participants came from around Great Britain, there was a preplanned focus on Oxfordshire and Buckinghamshire. Diversity was also limited, with only one participant from a minority ethnic background, again reflecting the general difficulty in recruiting ethnic minorities for research on mental health issues.^23^

### Implications

The discovery of self-harm is often a gradual process, with formal confirmation provided by an outside agency.^11^ Therefore, school or medical professionals may find themselves disclosing a child's self-harm to a parent and should be aware of the impact this may have. It is important to understand that parents may feel they should have known about their child's distress.^24^ Clearly, parents are in need of information about self-harm^20^ ^25^ and what to expect.^26^ Providing information about self-harm, and perhaps the opportunity to hear about the experiences of other families, may help carers to understand and manage the range of emotions they may feel and the impact on their social and family relationships.^12^ ^26^ ^27^ Clinicians, school staff and others working with families should be aware of the stigma associated with self-harm and avoid contributing to it by in any way appearing to blame parents for the child's problems.^25^ Parents can be encouraged to rely on family and friends for support and to take care of themselves, including seeking professional help (eg, for anxiety or depression). Clinicians should also be aware of the potential impact on siblings, who may themselves need help. Parents reported a desire for contact with other parents whose children self-harmed. The development of a support group for parents might serve the dual purpose of providing information and social support.^14^ ^20^ ^28^ In the workplace, employers should be encouraged to be flexible where possible. Parents who felt supported at work and able to take the time to help their child were more likely to stay in work, which could mitigate the financial consequences of self-harm and benefit the employer. Future research might include interviews with other family members, especially siblings. Speaking to the young people themselves would also give a more rounded picture of the effect of self-harm on the family.

## References

[1] Hawton K, Rodham K, Evans E Deliberate self-harm in adolescents: self report survey in schools in England. BMJ 2002;325:1207–11. 10.1136/bmj.325.7374.1207 12446536 PMC135492

[2] Brunner R, Kaess M, Parzer P Life-time prevalence and psychosocial correlates of adolescent direct self-injurious behavior: a comparative study of findings in 11 European countries. J Child Psychol Psychiatry 2014;55:337–48. 10.1111/jcpp.12166 24215434

[3] Madge N, Hewitt A, Hawton K Deliberate self-harm within an international community sample of young people: comparative findings from the Child and Adolescent Self-harm in Europe (CASE) Study. J Child Psychol Psychiatry 2008;49:667–77. 10.1111/j.1469-7610.2008.01879.x 18341543

[4] Hawton K, O'Connor RC, Saunders KEA Suicidal behaviour and self-harm. In Thapar A, Pine DS, Leckman JF, *et al*, eds. Rutter's Child and Adolescent Psychiatry 6th edn. Chichester: Wiley-Blackwell, 2015;893-910.

[5] Evans E, Hawton K, Rodham K In what ways are adolescents who engage in self-harm or experience thoughts of self-harm different in terms of help-seeking, communication and coping strategies? J Adolesc 2005;28:573–87. 10.1016/j.adolescence.2004.11.001 16022890

[6] Gratz KL Risk factors for deliberate self-harm among female college students: The role and interaction of childhood maltreatment, emotional inexpressivity, and affect intensity/reactivity. Am J Orthopsychiatry 2006;76:238–50. 10.1037/0002-9432.76.2.238 16719643

[7] Brophy M, Holmstrom R, . Truth hurts: report of the national inquiry into self-harm among young people. London: Mental Health Foundation, 2006.

[8] Geulayov G, Gunnell D, Holmen TL The association of parental fatal and non-fatal suicidal behaviour with offspring suicidal behaviour and depression: a systematic review and meta-analysis. Psychol Med 2012;42:1567–80. 10.1017/S0033291711002753 22129460

[9] Hawton K, Saunders KEA, O'Connor RC Self-harm and suicide in adolescents. Lancet 2012;379:2373–82. 10.1016/S0140-6736(12)60322-5 22726518

[10] Baetens I, Claes L, Onghena P Non-suicidal self-injury in adolescence: A longitudinal study of the relationship between NSSI, psychological distress and perceived parenting. J Adolesc 2014;37:817–26. 10.1016/j.adolescence.2014.05.010 25086458

[11] Oldershaw A, Richards C, Simic M Parents’ perspectives on adolescent self-harm: qualitative study. Br J Psychiatry 2008;193:140–4. 10.1192/bjp.bp.107.045930 18669999

[12] Byrne S, Morgan S, Fitzpatrick C Deliberate self-harm in children and adolescents: a qualitative study exploring the needs of parents and carers. Clin Child Psychol Psychiatry 2008;13:493–504. 10.1177/1359104508096765 18927136

[13] McDonald G, O'Brien L, Jackson D Guilt and shame: Experiences of parents of self-harming adolescents. J Child Health Care 2007;11:298–310. 10.1177/1367493507082759 18039732

[14] Rissanen M, Kylmä J, Laukkanen E Parental conceptions of self-mutilation among Finnish adolescents. J Psychiatr Ment Health Nurs 2008;15:212–18. 10.1111/j.1365-2850.2007.01214.x 18307650

[15] Coyne IT Sampling in qualitative research. Purposeful and theoretical sampling; merging or clear boundaries? J Adv Nurs 1997;26:623–30. 10.1046/j.1365-2648.1997.t01-25-00999.x 9378886

[16] Saunders MNK Choosing research participants. In: Symon CCG, ed.. The practice of qualitative organisational research: Core methods and current challenges. London: Sage, 2012:37–55.

[17] Hussain-Gambles M, Atkin K, Leese B Why ethnic minority groups are under-represented in clinical trials: a review of the literature. Health Soc Care Community 2004;12:382–8. 10.1111/j.1365-2524.2004.00507.x 15373816

[18] Ziebland S, McPherson A Making sense of qualitative data analysis: an introduction with illustrations from DIPEx (personal experiences of health and illness). Med Educ 2006;40:405–14. 10.1111/j.1365-2929.2006.02467.x 16635119

[19] Shenton AK Strategies for ensuring trustworthiness in qualitative research projects. Educ Info 2004;22:63–75.

[20] Raphael H, Clarke G, Kumar S Exploring parents’ responses to their child's deliberate self-harm. Health Educ 2006;106:9–20. 10.1108/09654280610637166

[21] Hughes ND, Locock L, Simkin S Making sense of an unknown terrain: how parents understand self-harm in young people. Qual Health Res. Published Online First: 2015 10.1177/1049732315603032 26369673

[22] Phares V, Lopez E, Fields S Are fathers involved in pediatric psychology research and treatment? J Pediatr Psychol 2005;30:631–43. 10.1093/jpepsy/jsi050 15772363

[23] Yancey AK, Ortega AN, Kumanyika SK Effective recruitment and retention of minority research participants. Annu Rev Public Health 2006;27:1–28. 10.1146/annurev.publhealth.27.021405.102113 16533107

[24] Rose H, Cohen K, Kinney C Mothers’ experiences of mental health services following their children's self-harm. Fam Sci 2011;2:196–202. 10.1080/19424620.2012.665379

[25] Rissanen ML, Kylmä J, Laukkanen E Helping adolescents who self-mutilate: parental descriptions. J Clin Nurs 2009;18:1711–21. 10.1111/j.1365-2702.2008.02672.x 19646117

[26] healthtalk.org. Self-harm: Parents’ experiences. Secondary Self-harm: Parents’ experiences 2014 http://www.healthtalk.org/peoples-experiences/mental-health/self-harm-parents-experiences/topics

[27] Smith J The parent's guide to self-harm: what parents need to know. Oxford: Lion Hudson, 2012.

[28] Power L, Morgan S, Byrne S A pilot study evaluating a support programme for parents of young people with suicidal behaviour. Child Adolesc Psychiatry Ment Health 2009;3:20 10.1186/1753-2000-3-20 19604392 PMC2717051

